# Supplementary material for: cfDNA Changes in Maximal Exercises as a Sport Adaptation Predictor
Source: Genes (Basel). 2021 Aug 12;12(8):1238. doi: 10.3390/genes12081238 (PMC8392318; doi:10.3390/genes12081238)
Supplement: Supplementary file 1 [file genes-12-01238-s001.zip › genes-1326479-supplementary.pdf]

Supplementary Results:

Table S1. Morphological parameters of the participants (n = 50).

Variables	Unit	Control (n = 25)		Athletes (n = 25)		p
		Mean ± SD	CI	Mean ± SD	CI	
WBC	10^9/L	5.80 ± 1.43	5.19-6.39	6.25 ± 1.46	5.61-6.87	0.29
RBC	10^{12}/L	5.14 ± 0.27	5.02-5.25	5.05 ± 0.27	4.93-5.16	0.26
Haemoglobin	g/dL	15.32 ± 0.79	15.00-15.64	15.32 ± 0.89	14.93-15.71	1.00
Haematocrit	%	43.99 ± 2.02	43.16-44.80	43.91 ± 2.15	42.98-44.84	0.90
MCV	fl	85.63 ± 3.21	84.33-86.93	87.02 ± 2.75	85.83-88.21	0.11
MCH	pg	29.85 ± 1.56	29.21-30.48	30.35 ± 1.17	29.84-30.86	0.22
MCHC	g/dl	34.84 ± 0.97	34.44-35.23	34.90 ± 0.59	34.63-35.15	0.81
RDW	%	12.44 ± 0.45	12.25-12.62	12.52 ± 0.56	12.27-12.76	0.57
Platelets	G/l	233.19 ± 41.86	216.28-250.10	233.65 ± 39.98	216.36-250.94	0.96
MPV	fl	11.13 ± 0.85	10.78-11.47	10.62 ± 0.78*	10.28-10.96	0.04
Neutrophils	G/l	2.99 ± 1.06	2.55-3.42	3.42 ± 1.40	2.81-4.02	0.23
Lymphocytes	10^9/L	1.99 ± 0.15	1.72-2.25	2.07 ± 0.43	1.88-2.26	0.60
Monocytes	109/L	0.54 ± 0.15	0.48-0.60	0.55 ± 0.12	0.49-0.59	0.92
Eozynocytes	109/L	0.19 ± 0.17	0.12-0.26	0.15 ± 0.09	0.11-0.19	0.31
Basophils	%	0.04 ± 0.02	0.03-0.05	0.04 ± 0.01	0.03-0.04	0.53
Glucose	mg/dl	91.35 ± 9.88	87.35-95.33	88.70 ± 6.08	86.06-91.32	0.27
T. CHOL	mg/dl	149.81 ± 25.76	139.39-160.21	152.74 ± 29.16	140.12-165.34	0.71
HDL	mg/dl	45.92 ± 11.49	41.27-50.56	54.70 ± 11.72*	49.62-59.76	0.01
Triglyceride	Mg/dl	71.38 ± 25.83	60.94-81.82	68.09 ± 28.23	55.87-80.29	0.67
LDL	Mg/dl	87.92 ± 21.74	79.14-96.70	84.22 ± 22.26	74.58-93.84	0.56
non-HDL	Mg/dl	101.31 ± 24.41	91.44-111.16	97.91 ± 25.88	86.71-109.10	0.64
Iron (Fe)	µg/l	121.73 ± 56.11	99.06-144.39	132.17 ± 59.70	106.35-157.99	0.53

Note: WBC, white blood cells; RBC, red blood cells; MCV, mean corpuscular hemoglobin concentration; MCH, mean corpuscular haemoglobin; MCHC, mean corpuscular hemoglobin concentration; RDW, red cell distribution width; MPV, mean platelet volume; T. CHOL, total cholesterol; HDL, high-density lipoprotein; TG, triglyceride; LDL, low-density lipoprotein level.
*significant difference vs control group at $p < 0.05$

Table S2. Maximal anaerobic effort (double repeated WAnT) performance of the participants.

**Variables**	**Unit**	**Control (n = 25)**		**Athletes (n = 25)**		**p**
		**Mean ± SD**	**CI**	**Mean ± SD**	**CI**	
**Relative peak power of the 1st WAnT**	W/kg	9.97 ± 0.92	9.59-10.34	10.58 ± 1.08*	10.13-11.03	0.03
**Relative mean power of the 1st WAnT**	W/kg	7.88 ± 0.66	7.61-8.15	8.50 ± 0.54*	8.27-8.72	< 0.01
**Relative peak power of the 2nd WAnT**	W/kg	7.35 ± 0.65	7.08-7.60	7.96 ± 0.69*	7.67-8.25	< 0.01
**Relative mean power of the 2nd WAnT**	W/kg	5.58 ± 0.49	5.38-5.78	6.20 ± 0.56*	5.97-6.43	<0.01

**Change of relative peak power**	W/kg	2.63 ± 0.98	2.22-3.02	2.62 ± 1.16	2.13-3.10	0.98
**Change of relative mean power**	W/kg	2.29 ± 0.64	2.03-2.55	2.29 ± 0.60	2.04-2.54	0.99

Note: WAnT, Wingate Anaerobic Test, *significant difference vs control group at $p < 0.05$

Table S3. Maximal aerobic effort (treadmill running till exhaustion - Bruce protocol) performance of the participants.

**Variables**	**Unit**	**Control (*n* = 25)**		**Athletes (*n* = 25)**		**p**
		**Mean ± SD**	**CI**	**Mean ± SD**	**CI**	
**Maximal oxygen uptake**	ml/min/kg	53.99 ± 5.28	51.75-56.22	62.23 ± 5.82*	59.72-64.75	< 0.01
**Maximal ventilation**	L/min	146.45 ± 23.42	136.56-156.35	159.78 ± 16.30*	152.73-166.83	0.03
**Maximal RER**	VCO_2/VO_2	1.16 ± 0.07	1.13-1.19	1.15 ± 0.05	1.13-1.17	0.53
**Maximal heart rate**	beats/min	189.04 ± 14.11	183.08-195.00	190.56 ± 11.39	185.63-195.49	0.68

Note: RER, the ratio of ventilation CO_2 expired to O_2 consumed, *significant difference vs control group at $p < 0.05$

Table S4. Two-way (2 groups x 4 repeated measures) ANOVA of serum cell free DNA (cfDNA) concentration induced by maximal effort in training and control group.

**Effort**	**Effect**	**F**	**df**	***p*-value**	**Effect size (η^2)**	**Post-hoc outcome**
**Anaerobic**	Group	9.25	1, 48	<0.01	0.17	C < A
	RM	7.38	1, 48	<0.01	0.13	II > I, IV
	Group × RM	4.59	3, 144	<0.01	0.10	C-I > C-IV<br>A-II > A-I, A-IV<br>A-II > C-II
**Aerobic**	Group	1.08	1, 48	0.30	0.01	
	RM	4.63	1, 48	<0.01	0.03	II, III > I, IV
	Group × RM	3.44	3, 144	0.02	0.08	C-III > C-IV

Note: RM, repeated measures; C, controls; A, athletes; I, baseline; II, up to 5 minutes after the effort; III, 30 minutes after the effort; IV, 60 minutes after the effort

Table S5. Two-way (2 groups x 2 types of effort) ANOVA analysis of cfDNA change from baseline to up to 5 min after and 30 min and 60 min after maximal anaerobic and aerobic effort in training and control groups.

**Variable**	**Effect**	**F**	**df**	***p*-value**	**Effect size (η^2)**	**Post-hoc outcome**

**cfDNA change immediately after maximal effort**	Group<br>Effort<br>Group × Effort	6.81<br>0.40<br>4.11	1, 48<br>1, 48<br>1, 48	0.01<br>0.12<br>0.05	0.014<br>0.01<br>0.08	C < A<br><br>A- MAnE > C- MAnE
**cfDNA change 30 min after maximal effort**	Group<br>Effort<br>Group × Effort	1.26<br>0.02<br>2.75	1, 48<br>1, 48<br>1, 48	0.26<br>0.87<br>0.10	<0.01<br><0.01<br>0.02	
**cfDNA change 60 min after maximal effort**	Group<br>Effort<br>Group × Effort	10.61<br>0.69<br>0.21	1, 48<br>1, 48<br>1, 48	<0.01<br>0.40<br>0.64	0.19<br>0.02<br><0.01	C < A

Note: C, control; A, Athletes; MAnE, maximal anaerobic effort.
